# Innovations in biomedical nanoengineering: nanowell array biosensor

**DOI:** 10.1186/s40580-018-0141-6

**Published:** 2018-04-11

**Authors:** YoungTae Seo, Sunil Jeong, JuKyung Lee, Hak Soo Choi, Jonghan Kim, HeaYeon Lee

**Affiliations:** 1 Department of Computer Science, Queens College of the City University of New York, Flushing, NY 11367 USA; 2 Department of Biology, Queens College of the City University of New York, Flushing, NY 11367 USA; 3 National Center for Efficacy Evaluation of Respiratory Disease Product, Korea Institute of Toxicology, Jeongeup, Republic of Korea; 4 Gordon Center for Medical Imaging, Department of Radiology, Massachusetts General Hospital and Harvard Medical School, Boston, MA 02219 USA; 5 Department of Pharmaceutical Sciences, Northeastern University, Boston, MA 02115 USA; 6 Mara Nanotech New York, Inc., New York, NY 10031 USA

**Keywords:** Nanobiosensor, Electrochemical sensor, Nanowell array electrode (NWA), Nanofabrication, Immunosensor, Electrochemical impedance spectroscopy (EIS), Biomedical nanoengineering systems

## Abstract

Nanostructured biosensors have pioneered biomedical engineering by providing highly sensitive analyses of biomolecules. The nanowell array (NWA)-based biosensing platform is particularly innovative, where the small size of NWs within the array permits extremely profound sensing of a small quantity of biomolecules. Undoubtedly, the NWA geometry of a gently-sloped vertical wall is critical for selective docking of specific proteins without capillary resistances, and nanoprocessing has contributed to the fabrication of NWA electrodes on gold substrate such as molding process, e-beam lithography, and krypton-fluoride (KrF) stepper semiconductor method. The Lee group at the Mara Nanotech has established this NW-based biosensing technology during the past two decades by engineering highly sensitive electrochemical sensors and providing a broad range of detection methods from large molecules (e.g., cells or proteins) to small molecules (e.g., DNA and RNA). Nanosized gold dots in the NWA enhance the detection of electrochemical biosensing to the range of zeptomoles in precision against the complementary target DNA molecules. In this review, we discuss recent innovations in biomedical nanoengineering with a specific focus on novel NWA-based biosensors. We also describe our continuous efforts in achieving a label-free detection without non-specific binding while maintaining the activity and stability of immobilized biomolecules. This research can lay the foundation of a new platform for biomedical nanoengineering systems.

## Introduction

**Fig. 1:**
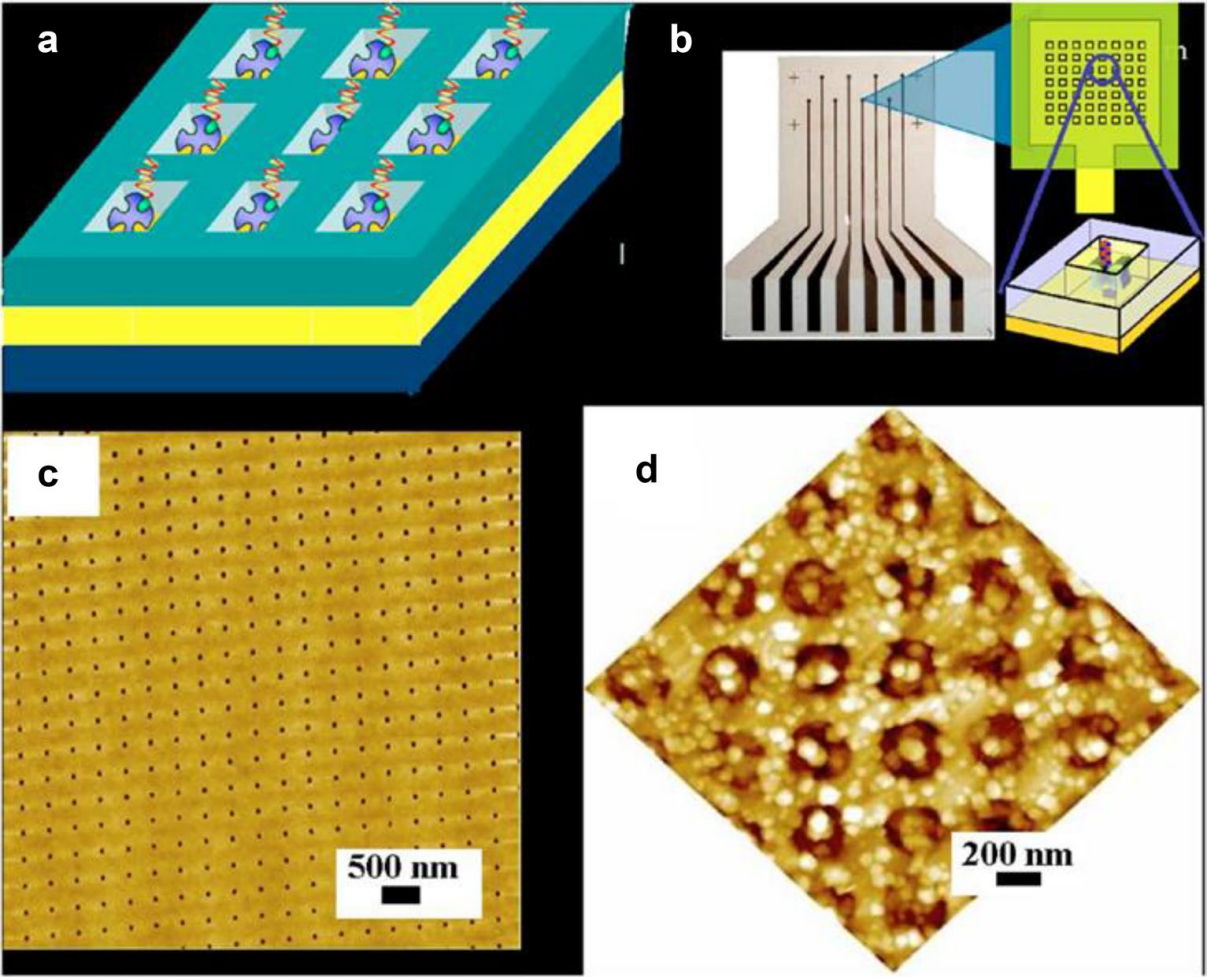
**a** Schematic diagram of the ONW array geometry on a gold electrode designed to minimize the unwanted, nonspecific binding or aggregation of biomaterials. **b** A well-oriented nanowell (ONW) array electrode composed of eight Au pads. The Au electrode on each pad was covered with a resist layer, i.e., a blocking layer. **c** An AFM image of an ONW array with a 200 × 200 probe grid on a gold electrode 800 μm in diameter. In fabricating the ONW array electrode, a 100 × 100 nm^2^ well was generated on the resist surface at 500 nm intervals using nanofabrication technology. **d** An AFM image of probe ssDNA immobilized by streptavidin-biotin on an ONW array. Although there are also bright spots on the resist layer, these DNA/streptavidin complexes do not contribute to the electrochemical signals

Protein micro- and nanoarrays have emerged as high-throughput screening tools for a variety of diagnostic assays, such as tissue engineering, pharmacology, and proteomics [1–10]. Miniaturized biosensors are currently being developed for the integration of electrical, optical, and physical measurements with fluid handling, [11–14]. It has been shown that the use of small quantities of sample can substantially improve the efficiency, speed, and accuracy of miniaturized detection technologies in a fast, high-resolution, low-cost manner [15–17]. For the advance of analytical biosensors, it is essential to develop a highly sensitive and reliable detection scheme for minute quantities of biomaterials. In some DNA assays, a biofunctional modification utilizing streptavidin molecules, coherently bound to a thiol-treated Au electrode, has been commonly used. Although this modification provides a significantly higher coverage of probe ssDNAs and longer-term stability [7, 8], non-specific bindings and aggregations among the DNA molecules significantly decrease its sensitivity. To fabricate more efficient and reliable bio-assays, it is essential to develop a method to immobilize probe ssDNAs with a more uniform coverage and in a geometric arrangement which minimizes non-specific bindings. Figure 1a shows our proposed nanowell array (NWA) structure, whose geometry has the potential to minimize the unwanted, non-specific binding or aggregation of probe ssDNAs. In this geometry, most of the Au electrode area is covered with a blocking layer such that only the nanosized gold surface is exposed to the open space above the NWA. The depth and width of the nanosized well can be specified to allow for only a few streptavidin molecules to enter inside the NWA and to become bound with the thiol-treated Au surface. Since one streptavidin molecule can bind with only one biotinylated oligonucleotide, [18] this NW array geometry would limit, to a small number, the amount of probe ssDNA molecules which stand inside each NW. Note that this geometry can easily be adapted to numerous other forms of biosensors [18–28].

Nanoarrays, which consist of probe biomolecules immobilized on a chemically modified surface, have attracted much attention for the development of nanobiosensors. However, there is no simple nanometric system that can commonly be used for high-selectivity nanobiosensor applications. This NWA structures with functional materials at the nanometer scale have become more important as technology advances and the paradigm shifts from single material and 2D planar structures to complex compounds and 3D architectures. It allows for the fabrication of unique and integrated devices such as gas sensors [29], optical photonic crystals [30], fluidic devices [31], stretchable electronics [32], ring shapes with ferromagnetic materials [33], and so on. Especially, nanoimprint lithography (NIL) is a promising technique that can produce low-cost, large-area fabrication, high throughput [34, 35]. However, pattern resolution is primarily determined by the mold and E-beam lithography (EBL) or Focused ion-beam lithography (FIB), and NIL cannot overcome the resolution limits of the patterning tools and is often limited by the type of materials [36–38]. NIL was used to fabricate protein nanoarrays with an inert poly ethylene glycol (PEG) polymer as the resist material. The most widely used inert materials are uncharged PEG-based polymers [39, 40] and self-assembled monolayers (SAMs) [41–43], but the latter is incompatible with the NIL process owing to difficulties in direct imprinting. We developed a very effective and widely applicable method for fabricating nanopatterns of a PEG hydrogel for protein NWA.

Construction of such NWAs on Au electrode with controlled dimension and density could allow for quantitative analysis on the level of single lipid vesicle with more reaction sites and low signal-to-noise (S/N) ratio. Non-specific binding has been a hurdle to most label-free detection methods since many proteins present in a biological sample adhered to the surface non-specifically, giving false positives. Strategies to overcome this problem involve specific chemistries, which limit non-specific binding, but they do not completely eliminate non-specific adsorption [29, 30, 44–46]. Thus, it would be of great benefit to develop a NWA electrode with controlled geometry and density that can capture individual liposomes without non-specific binding. To block non-specific binding, UV-curable PVA hydrogel, which acted as an inert barrier against non-specific adsorption of liposome was used so liposome only can bind to Au exposed area.

Electrochemical impedance spectroscopy (EIS) is commonly used for label-free detection of analytes such as proteins, DNA and peptides. EIS based immunosensors have high sensitivity and are relatively simple to operate in comparison to other immunological methods that involve optical or piezoelectric instrumentation. EIS-based immune sensors typically utilize antigen–antibody immune layers that are very thin and have low electric permittivity. Molecular binding is detected by the interruption of the faradaic current at the electrode, which generates an impedance signal [31, 32, 47]. In the impedance system, we measure the change in impedance at the electrode interface at the double layer and in solution. EIS can transduce these electrode boundary phenomena to electric signals.

In addition, we developed a highly sensitive immunosensor for quantitative detection of various proteins using wafer-scale nanowell array (NWA) electrodes. Wafer scale fabrication methods have low throughput and are limited to small areas [33–36]. Therefore, in this study, we used a krypton-fluoride (KrF) stepper semiconductor process with a wavelength of 248 nm for the fabrication of NWA electrodes on 6-inch wafer. This wafer-scale fabrication method of nano-patterns has rapid, high-throughput and is highly reliable for the fabrication of NWA biosensor.

## Nanoengineering of NWA structure

### 3D Au nanobox arrays

**Fig. 2:**
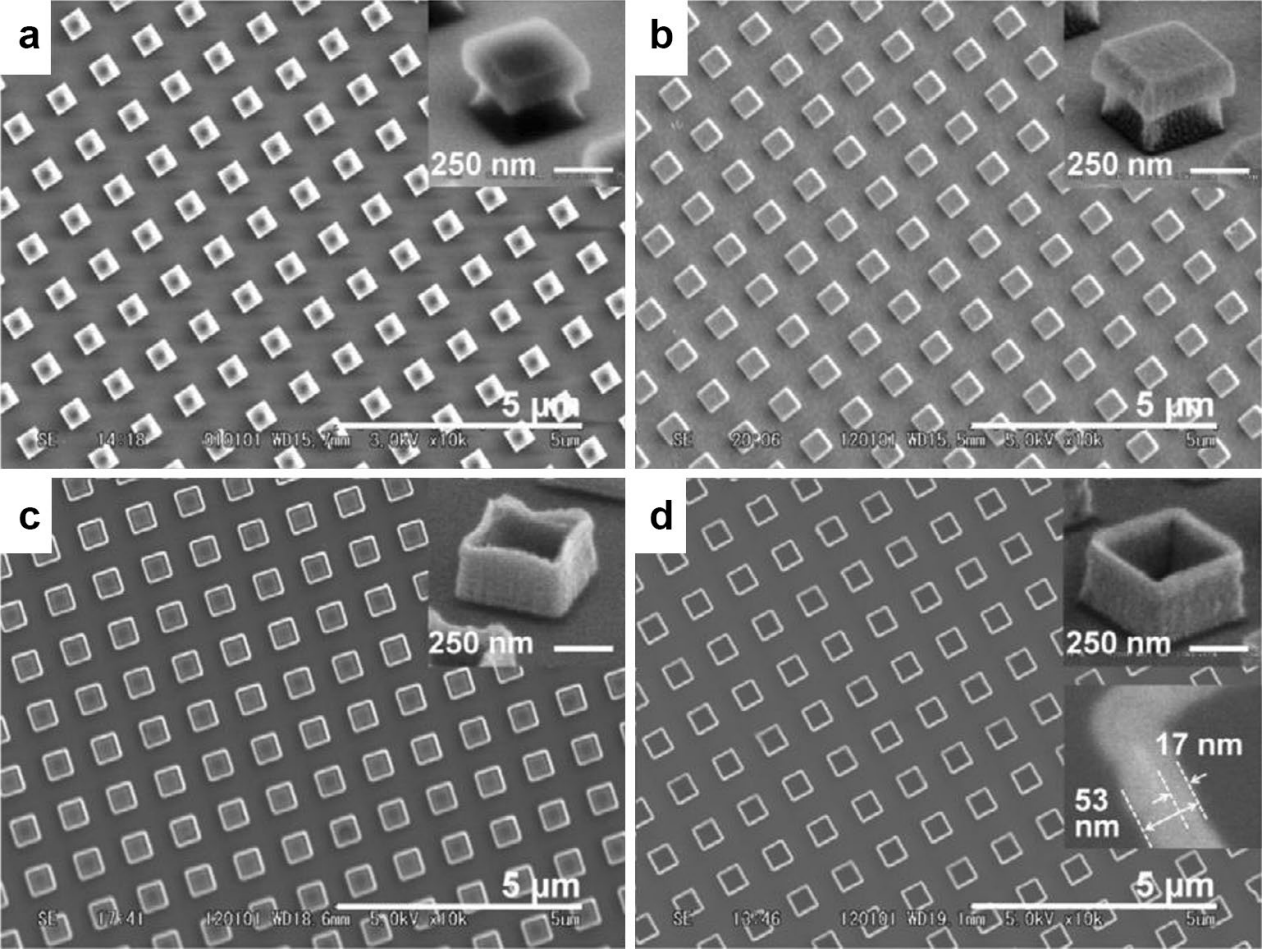
SEM images after **a** the RIE process, **b** 3 min of Au deposition, **c** 5 min of ion milling, and **d** 10 min of piranha cleaning. The bilayer polymer was imprinted by UV-NIL with a 400 nm square pattern. The thicknesses of the bending zone and undercut after RIE were around 53 and 17 nm, respectively. The insets represent 60°-tilted SEM images [48]

Figure 2a shows a top-view SEM image of 400 nm square polymer patterns after the two-step RIE process with the CF_4_ and O_2_ plasma. The top layer was almost rectangular, but the bottom layer showed tapered structures due to an undercut that occurred during the O_2_ plasma etching. The total pattern height measured by AFM was ∼ 350 nm after the two-step plasma etching. During the Au deposition by sputtering for 3 min, the patterns did not change and showed corners and sidewalls that were clearly coated (Fig. 2b). Note that Au was uniformly coated all around the top and bottom layers. It is assumed that the sputter system has a large deposition angle and good step-coverage, which can consistently coat complex sidewall structures. After ion milling for 5 min, the outside and top of the pattern areas were removed, and only the Au sidewall structures (or Au boxes) remained on the substrates (Fig. 2c). An ion milling process is a good anisotropic etching process but lacks selectivity, which means it is difficult to remove the resists completely. Dry cleaning methods can be more effective than wet methods in removing polymer resists sticking on the inside of high aspect-ratio nanostructures [37, 38, 48, 49]. After piranha cleaning for 10 min, clear square Au box arrays, empty inside, were obtained (Fig. 2d). The observed thicknesses of the bending zone and undercut were 53 nm and 17 nm, respectively.

**Fig. 3:**
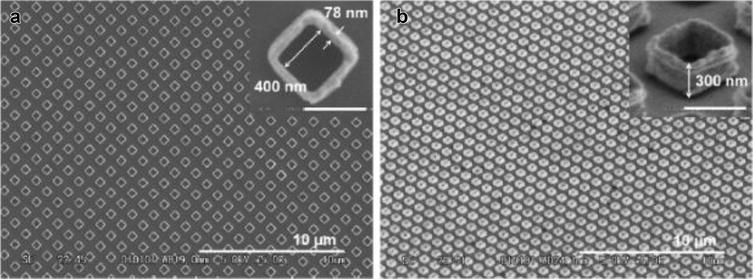
**a** Top and **b** 60°-tilted SEM images of large-area integrated Au nanobox arrays with a 400 nm inner square. The bilayer polymer was imprinted by UV-NIL with a 400 nm square pattern and subjected to 5 min of Au deposition, 5 min of ion milling, and 10 min of piranha cleaning. The scale bar of the insets is 500 nm [48]

Figure 3 shows top and 60°-tilted SEM images of large area integrated Au box arrays with an inside square of 400 nm, height of 300 nm, and a bending-zone thickness of 78 nm, after processing under the same conditions as outlined above, except for a 5 min of Au deposition. This method fabricates over 4 million nano-Au boxes at a time and shows a high reproducibility after repetition tests with no defects such that this process can be potentially scaled up as a practical technology. Furthermore, a simple cooking process with an automated machine will lower the potential barriers for commercial products.

**Fig. 4:**
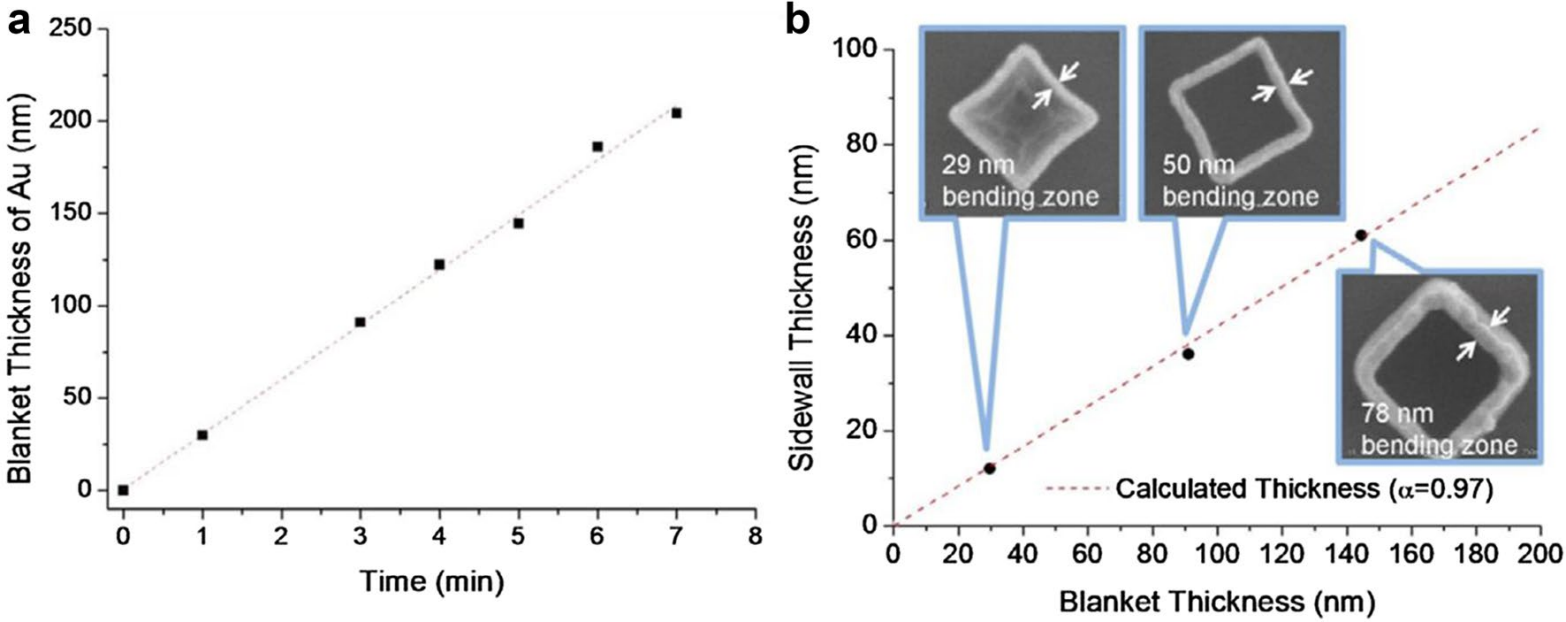
Changes of **a** blanket thickness as a function of deposition time and **b** sidewall thickness as a function of blanket thickness using a 400 nm square pattern. The dashed line indicates the calculated sidewall thickness [48]

The blanket thicknesses (normal growth on substrates without patterns) were experimentally plotted as a function of time (Fig. 4a), and the experimental results of the sidewall thickness were plotted as a function of the blanket thickness (Fig. 4b). Obviously, the blanket thickness increased linearly as the deposition time increased, and the sidewall thickness almost increased linearly with increasing blanket thickness.

**Fig. 5:**
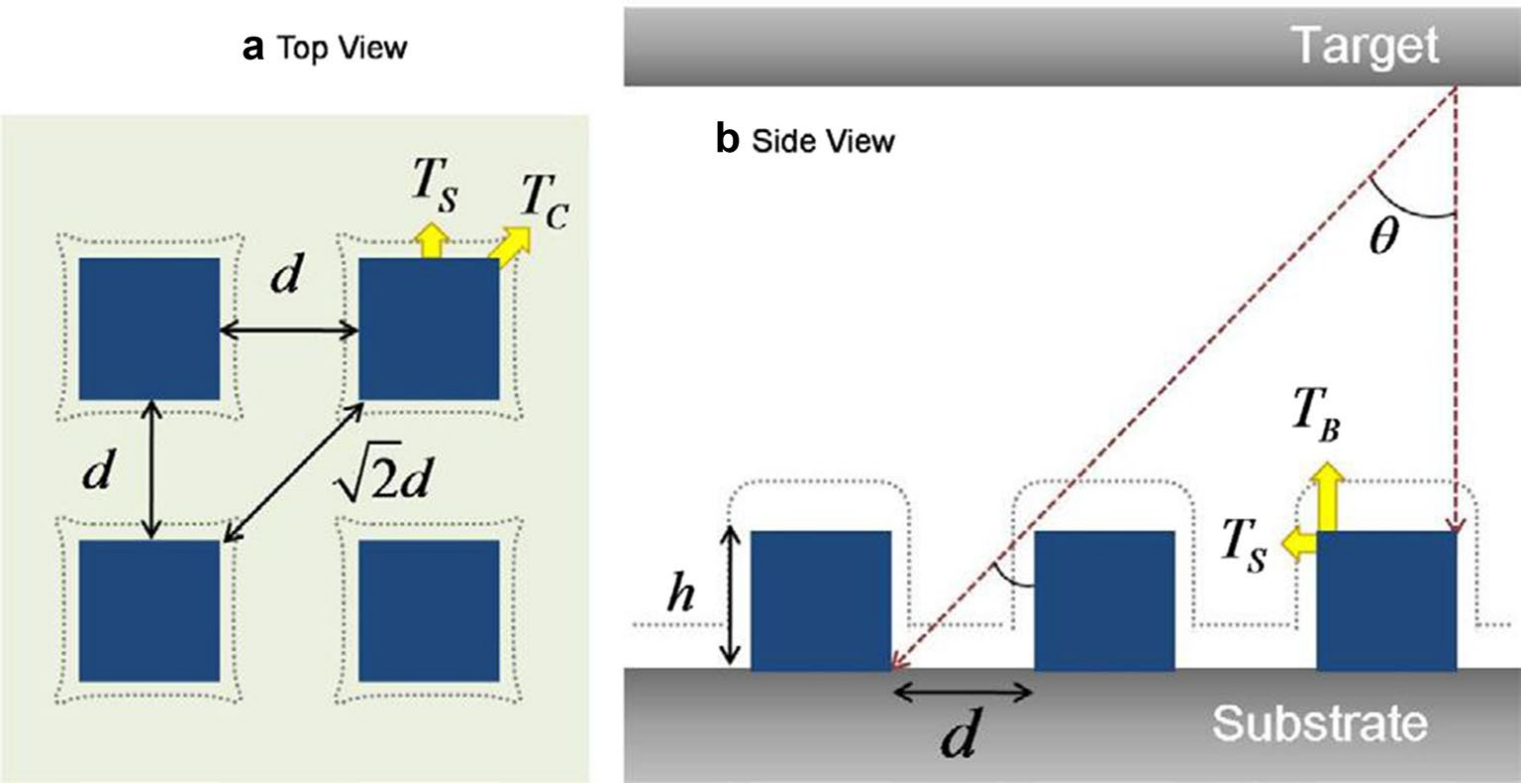
Schematic representation of geometric factors considered when calculating sidewall thickness [48]

Figure 5 details the geometric factors used to calculate this relationship. Assuming that a sputter deposition with a mean free path is small compared to the reactor dimensions, the incoming metal atoms above the target are isotropic, the sidewall thickness is small compared to the pattern distance and sidewall growth occurs on both sides homogeneously and in a manner proportional to blanket thickness [50], the simple equation is given by:

$$T_{\text{S}} = \frac{{\alpha T_{\text{B}} }}{2}\sin \theta$$

**Fig. 6:**
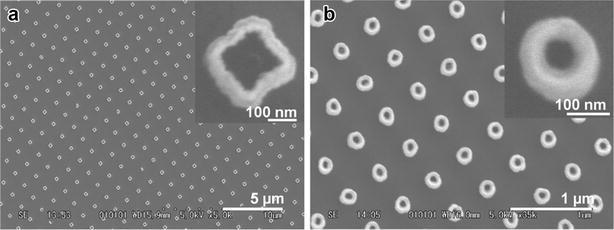
SEM images of different 3D Au hollow nanostructures. **a** Au nanobox arrays with ∼ 160 nm inner squares and **b** Au hollow pillar arrays with ∼ 65 nm inside pores were made using 200 nm square and 100 nm circular pattern molds, respectively [48]

Extending our experiments, smaller-sized nanobox and hollow nanopillar arrays were fabricated using different molds with the same process with 3 min of Au deposition. Figure 6a shows the nanobox structure with inner squares measuring ∼ 160 nm, and Fig. 6b shows the nanopillar structure with inside pores measuring ∼ 65 nm, which were fabricated by using a 200 nm square and a 100 nm circular hole-pattern mold, respectively. This demonstrates that integrated 3D hollow nanostructures can be fabricated directly without difficulty by applying the same process and conditions for smaller patterns.

### Double oxide deposition and etching (DODE) lithography

**Fig. 7:**
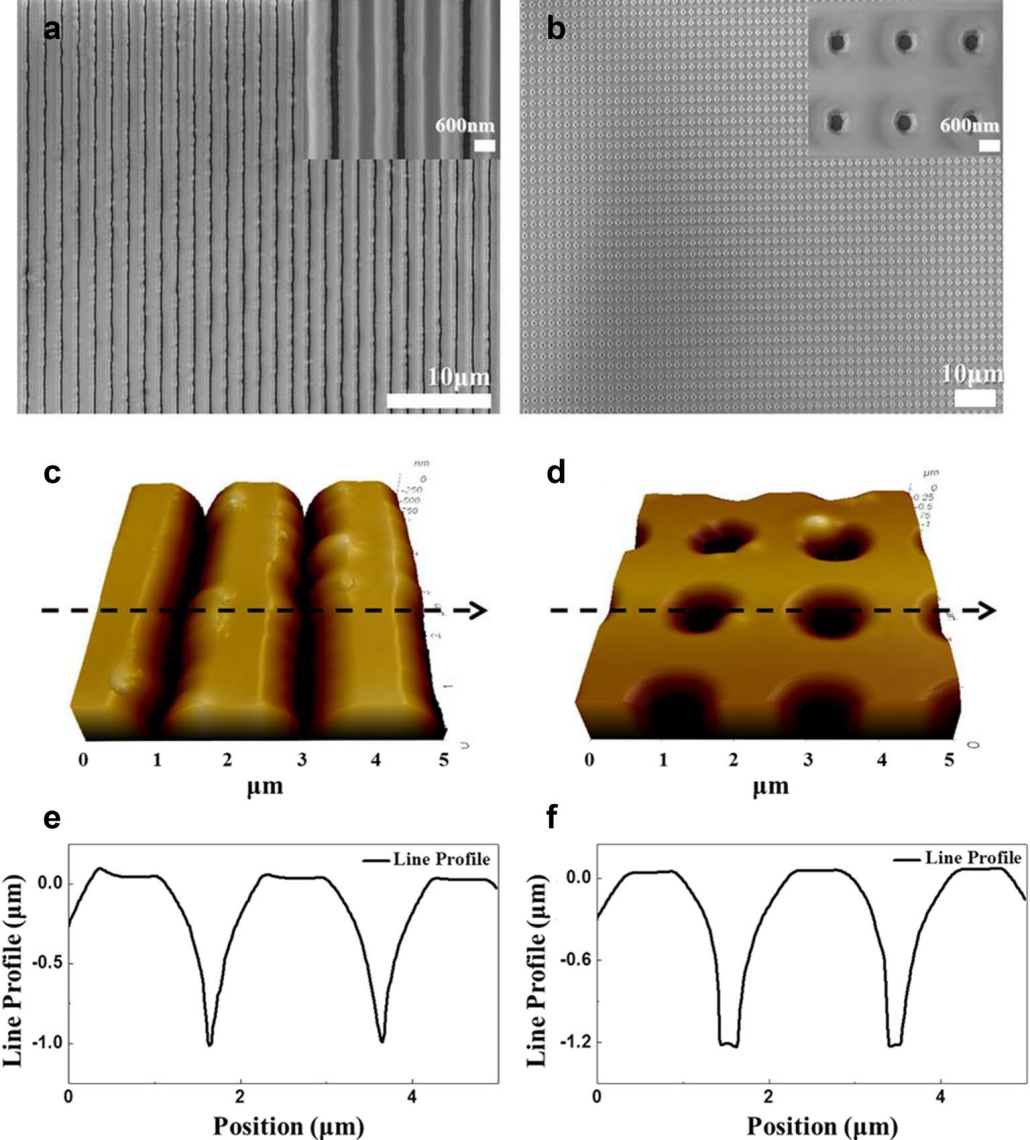
FE-SEM images of large-area of **a** nanotrench and **b** nanowell array and AFM images of **c** nanotrench and **d** nanowell arrays after the DODE lithography process; the inset shows high-magnification images of nanopatterns; and line profiles of each **e** nanotrench and **f** nanowell patterns measured from AFM images; high aspect ratios greater than 20:1 were obtained [51]

Figure 7a, b are SEM images of nanotrench and nanowell array patterns, respectively, on wafer-scale nanostructures. The minimum patterning sizes of a 50 nm linewidth for a nanotrench and a 400 nm diameter for a nanowell array were obtained using the developed DODE lithography method. In addition, we can manufacture uniform nanostructures with high aspect-ratio. In this study, a 1.5-µm-thick second SiO_2_ layer was deposited onto the microstructure of the 500-nm-thick first SiO_2_ layer to obtain sub-100 nm nanopatterns and structures with high aspect-ratio. After removal of 1 µm-thick residue SiO_2_ layer using dry etching process, 1 µm height of SiO_2_ nanostructure remained with 50 nm of nanopattern. The nanostructure surface morphologies for the 50 nm nanotrench patterns and the 400 nm nanowell array patterns were analyzed using atomic force microscopy (AFM), as shown in Fig. 7c, d, respectively. The line profile of a 1-µm deep nanowell with a 50 nm linewidth was measured using the AFM (Fig. 7e).

**Fig. 8:**
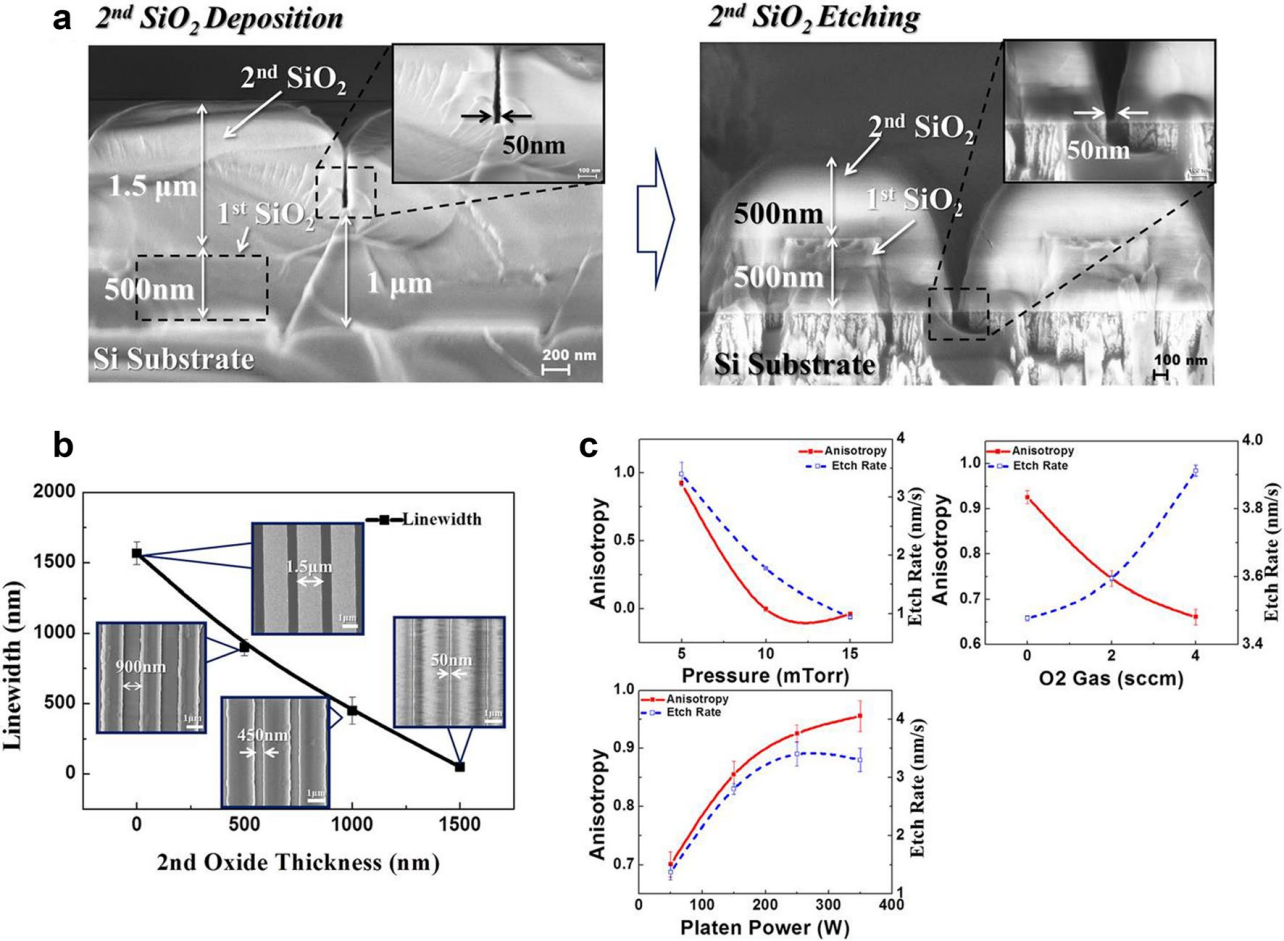
The DODE nanolithography process is demonstrated. **a** Cross sectional SEM images of a 50 nm of grooved nanostructure prepared via the DODE lithography process; a nanopattern on a residual SiO2 layer after isotropic deposition of a second SiO2 layer (inset shows high magnification image of the nanopattern) and SiO2-based grooved nanostructure on a Si substrate after anisotropic dry etching of a second SiO2 layer (inset shows high magnification image of the nanostructure). The nanopattern was obtained through 2nd SiO2 deposition process using PECVD on lithographically patterned microstructure. Then, the residual layer under nanopattern was removed using ICP etching process to define high aspect-ratio nanostructure. **b** Linewidth in microstructure is reduced according to the increased thickness of the second SiO2 layer by isotropic thin film deposition process (inset shows the SEM images of the 2nd SiO2 deposited nanopatterns). The linewidth of micropatterns was gradually decreased as the increase in 2nd SiO2 thickness up to 50 nm of nanopatterns. **c** Investigations of the anisotropic plasma etching process in terms of the effects of pressure, oxygen gas flow rate, and platen power. Anisotropy (A) increased as decrease in pressure and O2 gas flow rate, and increase in platen power (straight and dash lines represent anisotropy and etch rate, respectively) [51]

The DODE lithography process for high aspect-ratio nanostructure manufacturing with sub-50 nm of linewidth is depicted in Fig. 8. The cross-sectional FE-SEM (field emission scanning electron microscopy) images in Fig. 8a show the 50 nm linewidth of grooved nanostructures fabricated using the DODE lithography process. The microstructures have the 1.5 µm of micropatterns and the 500 nm of height, and then 1.5 µm of 2nd SiO_2_ layer was deposited subsequently to obtain nanopatterns. The total height of the nanostructures and the residue layer were 2 µm and the 1 µm, respectively. The linewidth of micropatterns was decreased as a function of the thickness of the second SiO_2_ layer as shown in Fig. 8b. The SiO_2_-based microstructures on a Si substrate with a 1.5 µm micropattern dramatically decreased to sub-50 nm nanopatterns following the second SiO_2_ deposition accordingly with high step coverage. Thus, an increase in the sidewall thickness of microstructures caused a decrease in micropatterns producing the nanopatterns. Anisotropic plasma etching process is demonstrated in Fig. 8c by investigation of nanoscale trench plasma etching process. The mean free path of the plasma radicals can be modified by the plasma etching conditions. A low mean free path produces an isotropic etching profile with a lateral etch rate that is approximately equal to the downward etch rate. Therefore, optimized etching conditions are an important factor in obtaining high-mean-free-path plasma radicals for high aspect-ratio nanotrench [52, 53].

High aspect-ratio nanostructure fabrication techniques have been developed by Morton et al. [54–57]. Involved nanomold imprinting to transfer patterns on a substrate, sacrificial layers deposition for bulk silicon substrate etching and polymer replication processes, which cause the nanostructure manufacturing process being difficult, time-consuming, and expensive. However, the DODE technique can simply fabricate wafer-scale high aspect-ratio nanostructures with nanoscale patterns by isotropic oxide deposition and anisotropic oxide etching processes without e-beam lithography or nanoimprint lithography processes. In addition, the DODE method does not use a polymer resin as a masking layer in the dry etching process so the nanostructures fabricated using the DODE process can be used as a polymer-free nanotemplate for various nanoelectronic applications.

### Hydrogel NWA electrodes on Au substrate

**Fig. 9:**
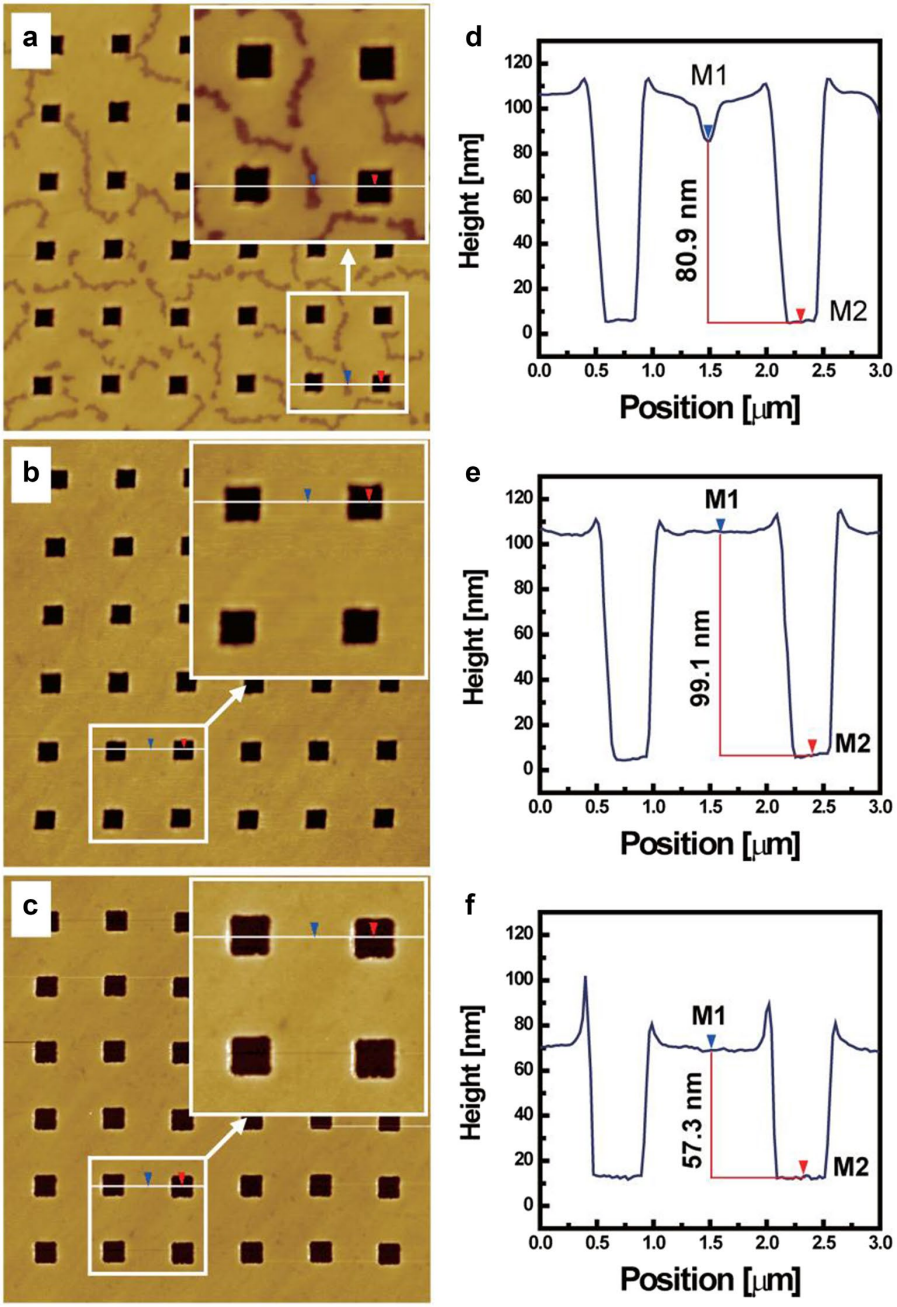
**a**–**c** Height and **d**–**f** cross-sectional TM-AFM images of 500-nm patterns of PEGDA575 hydrogel on gold substrates in air. **a**, **d** Mold-detached state after being prebaked at 80 °C for 10 min. **b**, **e** Molddetached state, and **c**, **f** O2 RIE finished state after being prebaked at 100 °C for 10 min [58]

Figure 9 shows height and cross-sectional tapping mode atomic force microscopy (TM-AFM) images of the 500-nm patterns of PEGDA575 hydrogel on gold substrates. Regarding the nanopattern of PEGDA575 prebaked at 80° for 10 min in cleanroom conditions, some dimples (20 nm in height) were observed on the surface of the PEGDA575 hydrogel, despite the nanopattern being created by UV-NIL (Fig. 9a, d). The dimples disappeared with an increase in the prebaking temperature to 100 °C for 10 min (Fig. 9b, e). This indicates that solvent retention is one of the reasons for the surface depression of PEGDA575 hydrogel. The imprinted depth (99.1 nm) of the PEGDA575 NW was almost the same as the mold height (100 nm), suggesting that the mold patterns were faithfully transferred by UV-NIL. The residual layers of PEGDA575 and BAC SAM in the NWs were successfully removed by O_2_ RIE (Fig. 9c, f). Compared to the PEGDA575 hydrogel, the PEGDA258 hydrogel has higher mechanical strength [59, 60]. However, we found that fabricating the nanopatterns of PEGDA258 hydrogel using UV-NIL was very difficult. Dimples were also present on the surface of the PEGDA258 hydrogel, but micropatterns larger than 3 mm were successfully replicated without dimples. Therefore, the PEGDA575 nanopatterns were used for protein nanoarrays throughout the experiments.

**Fig. 10:**
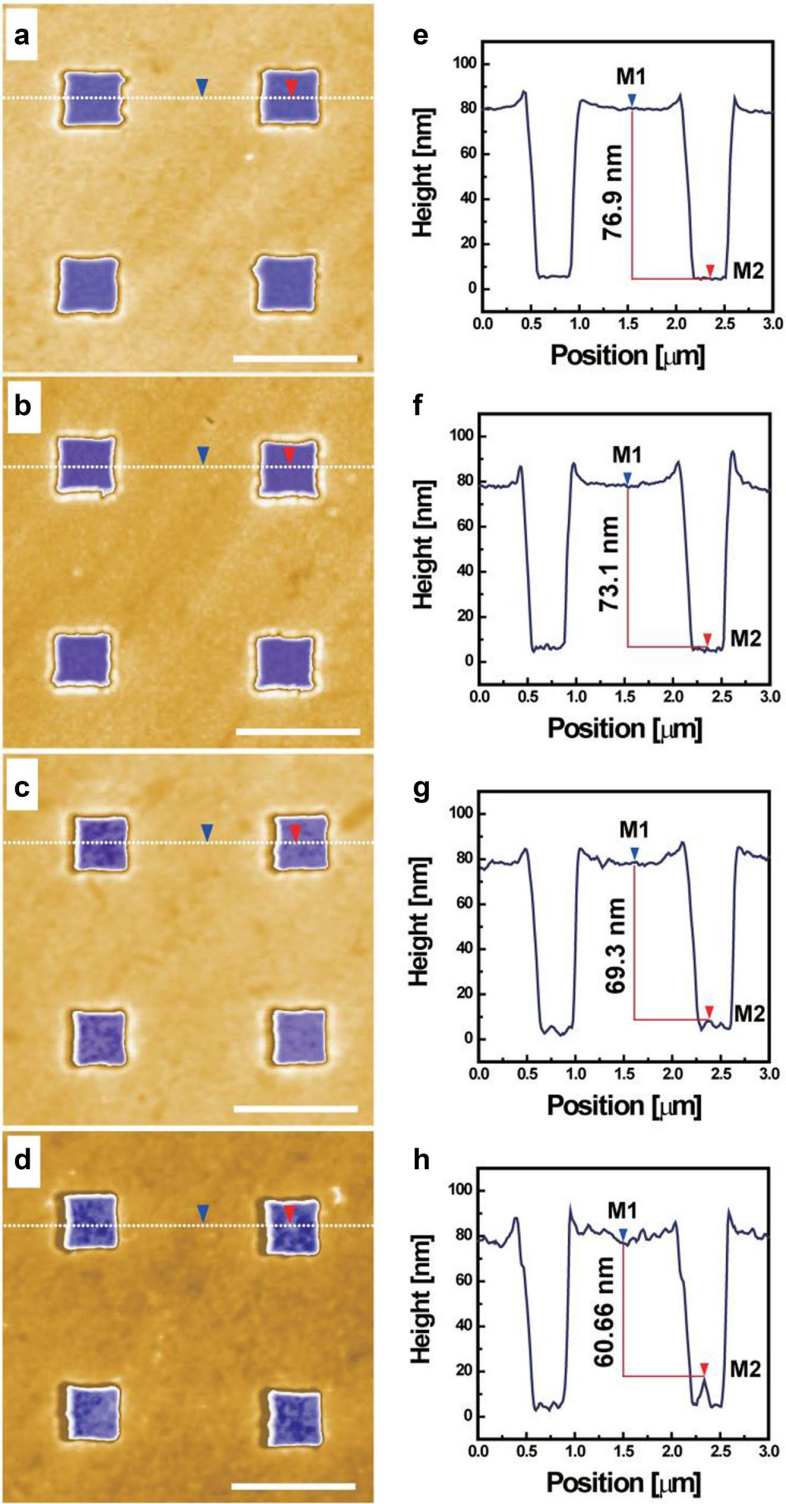
**a**–**d** Height and **e**–**h** cross-sectional TM-AFM images of the 500-nm patterns of PEGDA575 hydrogel on gold substrates in 10 mm PBS solution. **a**, **e** Hydrated state, **b**, **f** PEG mixed SAM-modified state, **c**, **g** SA-immobilized state, and **d**, **h** anti-bHSA-immobilized state. Scale bars: 1 μm [58]

**Fig. 11:**
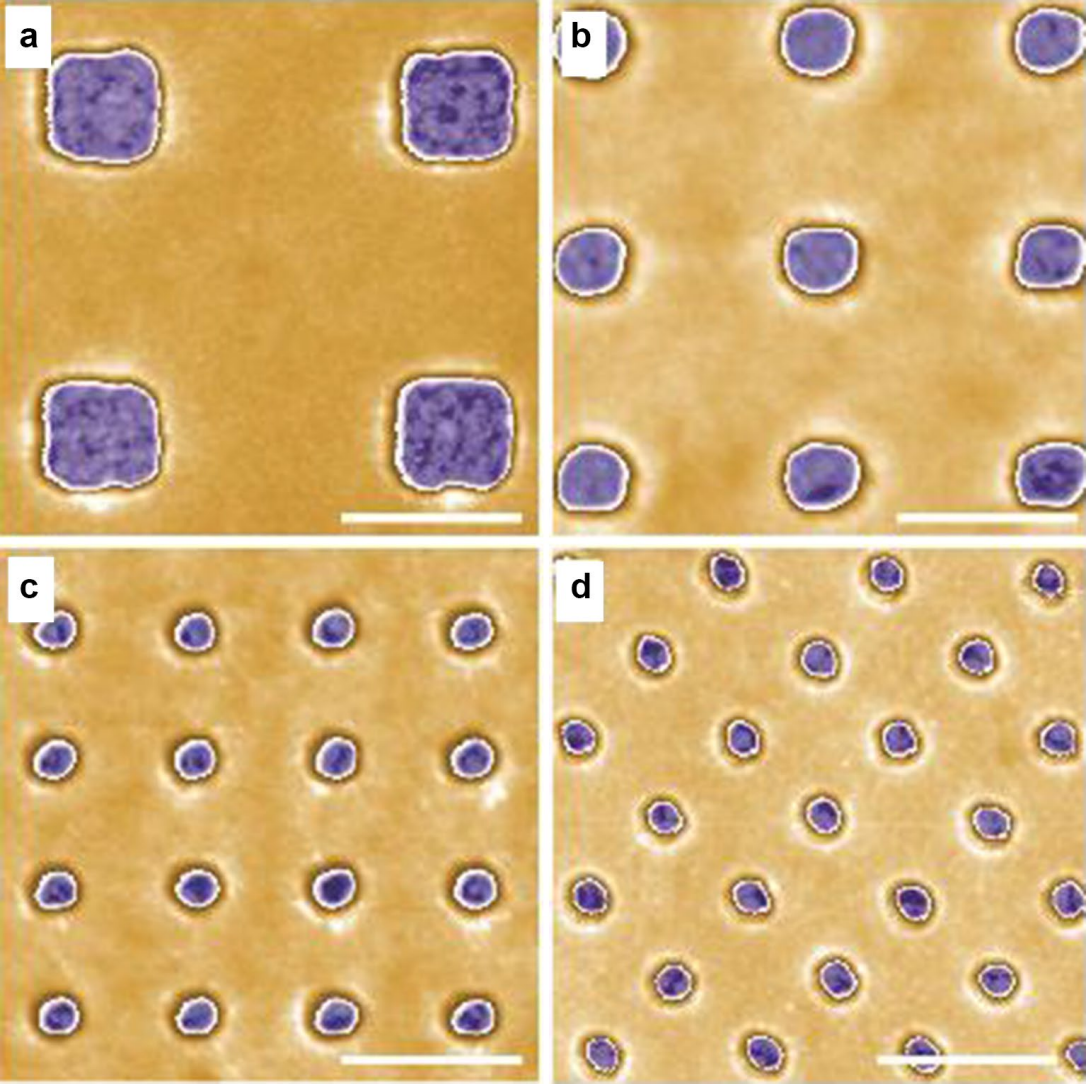
Height TM-AFM images of **a** 300 nm, **b** 200 nm, **c** 120 nm, and **d** 100 nm patterned PEGDA575 hydrogel on gold substrates at the anti-bHSA-immobilized state in air. Scale bars: 500 nm [61]

As shown in Fig. 10a, e, the NW depth of hydrated PEGDA575 increased by about 20 nm compared to dry PEGDA575 (Fig. 10c, f). However, neither surface roughness nor the largest lateral swelling possible was observed. In addition, after a period of up to 1 week in a 10 mm PBS solution, the nanopattern of the PEGDA575 hydrogel was not delaminated from the gold substrate, even though it was swollen (34% in height) by hydration. This result suggests that the acrylate groups of BAC on gold substrates are strongly bound to PEGDA groups by UV irradiation and that BAC is a suitable material for the adhesion of PEG-acrylate hydrogels on gold substrates. The NW depth of PEGDA575 hydrogel decreased by 3.8 nm compared to the hydrated state and mPEG-S/bPEG-S adsorbate was not found on the PEGDA575 surface. These results indicate that the gold substrate patterned with PEGDA575 hydrogel was selectively modified by the mPEG-S/bPEG-S mixed SAM. In the SA-immobilized state, NW depth decreased by 3.8 nm compared to the mixed SAM-modified state, indicating that the SA was confined to the NW of PEGDA575 hydrogel. The difference in NW depth between the anti-bHSA- and SA-immobilized states was 8.64 nm. Protein adsorbates were not observed on the PEGDA575 surface during the array process. To verify these results, the sizes of thiol-functionalized PEG, SA, and anti-bHSA in 10 mm PBS solution were measured as 3.9, 3.5, and 9.1 nm, respectively. The results suggest that the mPEG-S/bPEG-S mixed SAM and proteins were selectively and gradually self-assembled into the PEGDA575 hydrogel NWs on gold substrates. Figure 11 shows AFM height images of NWA of various sizes on gold substrates for the anti-bHSA-immobilized state in air. The periodic protein nanopatterns were only constructed in the PEGDA575 NWs. The contrast of arrayed proteins is clear for the 300-nm pattern and selective immobilization of anti-bHSA in the PEGDA575 NWs could also be observed for the sub-200-nm patterns, although the contrast was faint at the smaller NW size. Probe protein (anti-bHSA) nanoarrays with 100-nm feature size were accomplished, as shown in Fig. 11d. These results suggest the feasibility of constructing a protein nanoarrays with a feature size of sub-100 nm using UV-NIL.

### Wafer-scale fabrication of NWA

**Fig. 12:**
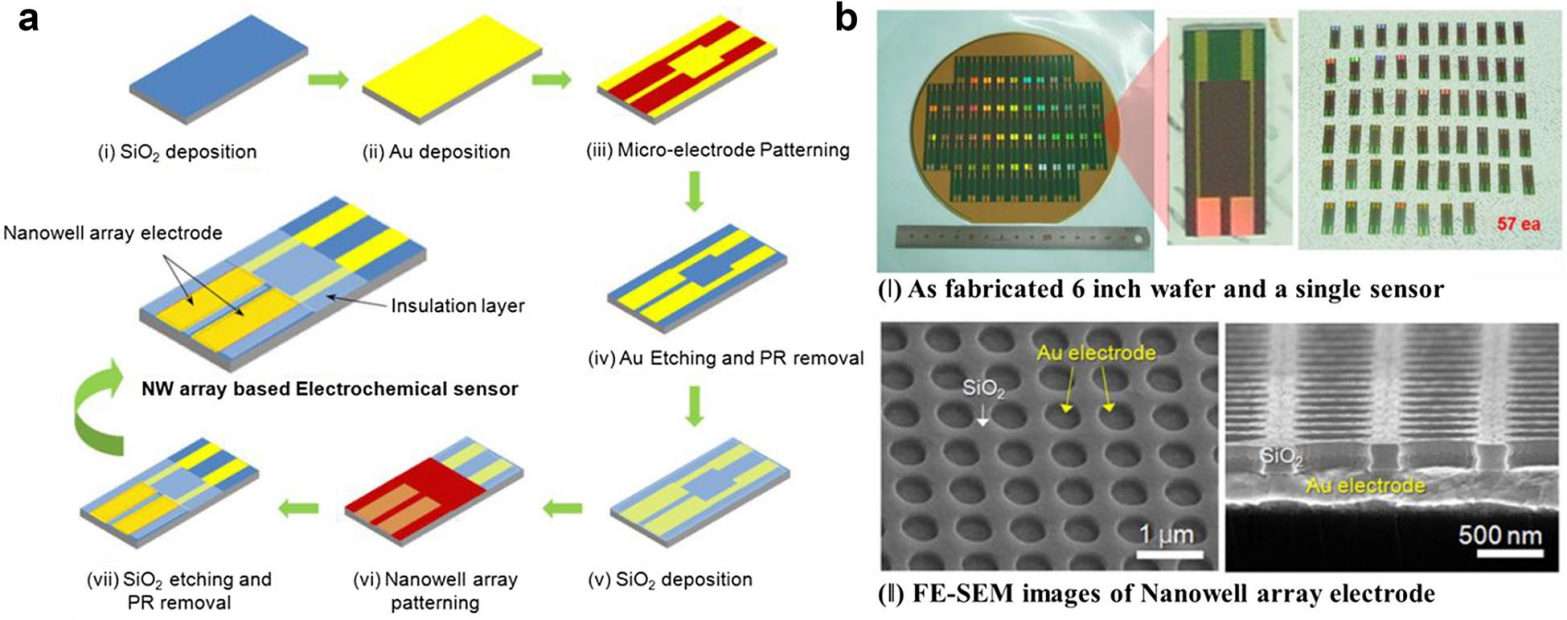
**a** Fabrication of NWA based electrochemical sensor. **a** simple and high throughput nano-process for industrial application. **b** Photograph of as-fabricated samples (top) and SEM images of NWA electrode (Bottom) [67]

Nanosized well structure at the molecular level was integrated by top down technology. We focused on a nanopatterning process using krypton-fluoride (KrF) stepper with a wavelength of 248 nm for fabricating NWA electrode in large area. E beam lithography (EBL) is good process to fabricate NWA however, EBL is low throughput method (5 wafers/h at less than 0.1 µm resolution). To overcome this limitation, KrF stepper was used because it has better overlay and alignment and high throughput (50–80 wafers/h) compared with EBL. A KrF stepper was used for nano-scale patterning of NWA sensor on 6-inch wafer after PR coating as shown in Fig. 12a. Figure 12b shows the 57 uniform and well-fabricated NWA electrodes on a 6-inch Si wafer fabricated by KrF stepper semiconductor process for industrial application. The size of a single NWA sensor was 21 mm × 10 mm. Each sensor consisted of two NWA with a size of 4 mm × 2 mm. Each NWA structure had a diameter of 500 nm with an interwell spacing of 200 nm.

It was confirmed that each NWA structure had a diameter of 500 nm with interspacing of 200 nm and a height of 200 nm. The NWA electrode, the counter electrode and reference electrode were combined into the electrode holder. The liquid reagents were treated to the electrode and electrochemical analysis was performed.

## Biomedical nanoengineering platform

### Enhancement of integrated electric nanobiosensor utilizing NWA geometry

To demonstrate its usefulness, we fabricated a NW array-based gene assay for EC sensing, which has attracted a great deal of attention due to its high sensitivity, low cost, and high portability [62–70]. Figure 1b displays an illustration of our experimental electrode, composed of eight Au pads. The thickness of the resist layer was varied between 150 and 200 nm. And, each NW was 50 nm in diameter and separated by 500 nm. The atomic force microscope (AFM) picture, in Fig. 1c, illustrates that arrayed 50 nm NWs were successfully fabricated. Inside the NWs, probe ssDNA molecules were immobilized. The diameter of each streptavidin molecule used in this study was approximately 10 nm [71]. The diameter of each streptavidin molecule used in this study was approximately 10 nm [71]. Therefore, the maximum allowed number of the streptavidin–biotinylated DNA within a 50 nm NW is less than 3. The actual number should be much less than 3, since the actual diameter of the exposed gold dot is smaller than 50 nm due to the cutting angle of the fabricated resist layer. To directly observe whether the ssDNA molecules could be immobilized within the NWs, we performed AFM measurements. Initially, we attempted to obtain good AFM pictures on 50 nm NW arrays, but failed (due to the NW size, which might be too small and deep for an AFM cantilever to enter the holes). Instead, we performed AFM measurements on a 200 nm NWAs. Figure 1d shows an AFM picture of a NWA electrode subsequent to the ssDNA immobilization process. There are several bright spots inside each NWA, which serves as indirect confirmation that several probe DNA/streptavidin complexes could be attached inside the NWA. There are also bright spots on the resist layer, but these DNA/streptavidin complexes would not contribute to the EC signals. Although this AFM picture was taken on the 200 nm NW array, it demonstrates that the NW geometry concept, proposed in Fig. 1c, could work for our 50 nm NW array.

### Electrochemical nanobiosensor: DNA

**Fig. 13:**
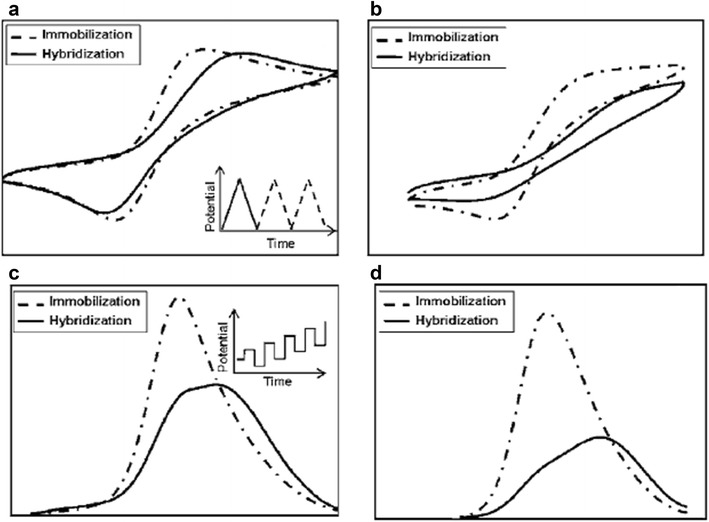
Electrochemical assay of label-free DNA protocol on an electrode utilizing **a** 200 μm diameter electrode **b** NWA with 50 nm diameter (number of 200 × 200). Electrochemical measurement (square-wave voltammograms and cyclic voltamogram were carried out in 10 mM K3Fe(CN)6 solutions at a scan rate of 100 mV/s: before immobilization (dotted line), and after hybridization (solid line) with a complementary target 21-mer oligonucleotide. Electrochemical response change between the redox current after immobilization (*Iim*) and after hybridization (*Ihy*) in the 200 μm electrode **a**, **b** and NWA electrodes **c**, **d**. The concentration of probe DNA and complementary target DNA was 50 μM/100 mL

Figure 13b illustrates EC response changes of the 50 nm NW array due to hybridization of label-free complementary DNA molecules. The hybridization was carried out at a potential of + 300 mV for 3 min. Owing to its anionic characteristics, the DNA could be hybridized onto the Au electrode by applying a positive bias [67]. The red and blue lines represent the square-wave voltammetry (SWV) data obtained after immobilization (I_im_) and hybridization (I_hy_), respectively. With the complementary DNA concentration of 50 μM in a mediator solution of K_3_Fe(CN)_6_, the electric signal changes (∆I/I_im_) due to hybridization fell by 63% for the array. This ∆I/I_im_ value was higher by about 20% than that for a 200 μm diameter Au electrode, shown in Fig. 13a. Since the active area for EC signals of the NW array electrode was 100 times smaller than that of the 200 μm diameter electrode, the change of ∆I/I_im_ represents a 20-fold enhancement in overall sensitivity. Our EC gene protocol, utilizing the NW array, also provided a very good reproducibility. Redox currents, checked with cyclic voltammetry (CV) measurements, were displayed in the insets of Fig. 13. The redox reaction, occurring in the K_3_Fe(CN)_6_ solution, was clearly demonstrated by the peaks in both the reduction and oxidation curves with a difference in peak potential (∆E_p_) of less than 15 mV.

### Electrochemical nanobiosensor: functional lipid vesicle

**Fig. 14:**
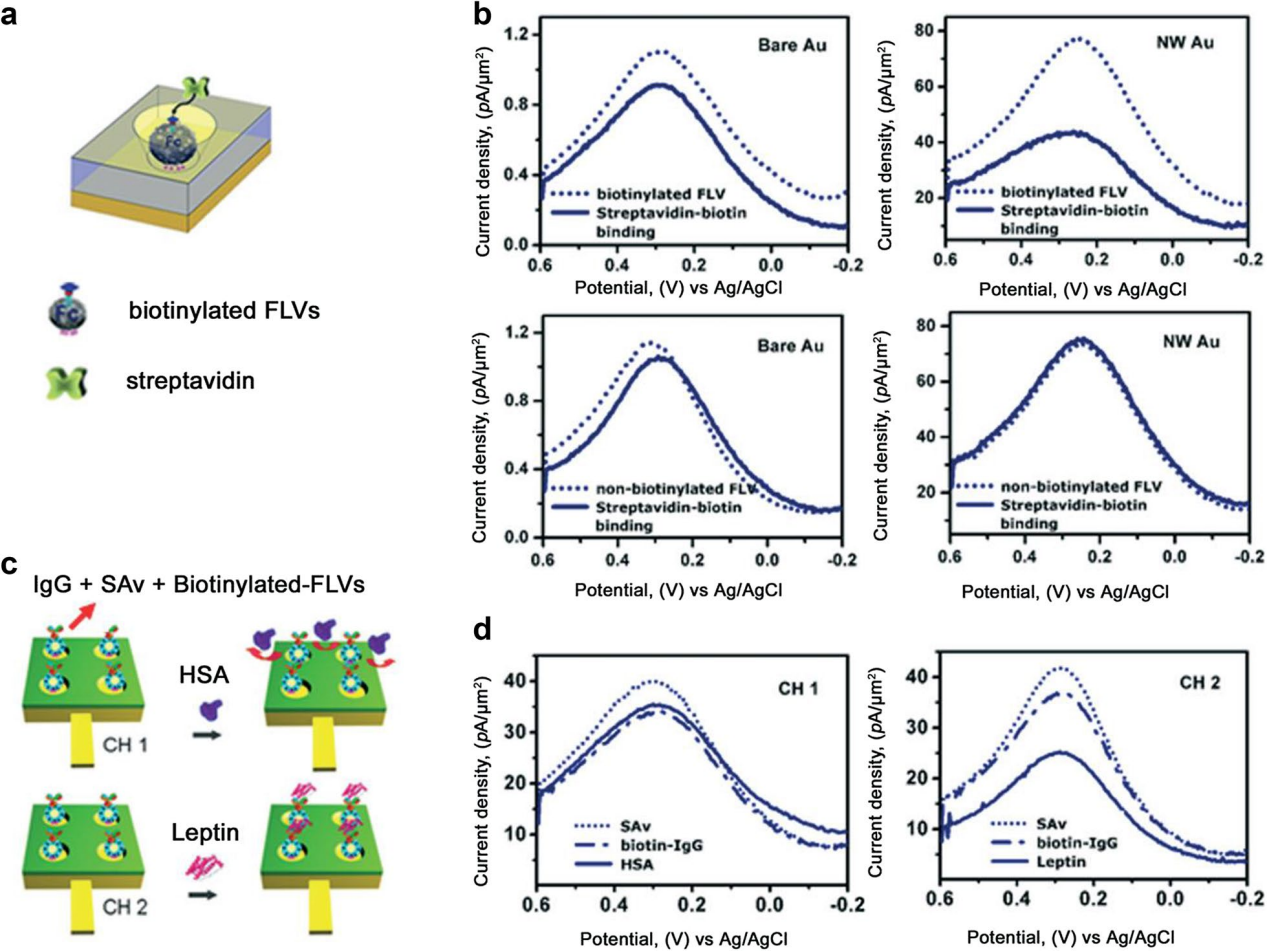
**a** A schematic illustration of the binding event between streptavidin and the captured biotinylated FLVs on NW electrode. **b** SWV measurements. **c** A detection scheme of leptin. **d** SWV measurements of current density after addition of the biotinlylated anti-leptin [75]

To evaluate potential applications of the site-selective deposition of single functional lipid vesicle (FLVs), we tested streptavidin binding to biotin on the FLVs surface. This coupling is widely studied for development of biosensor for its strong affinity (dissociation constant, *K*_d_ = 10^−15^ M) [72–74]. To claim that NW electrode can act as a restrictive surface for bioassays, the binding event should block the electron transfer to NW electrode (so called the shielding effect). Figure 14a illustrates the streptavidin–biotin interactions using biotinylated FLVs containing thiol, ferrocence and PEG moieties on the surface of FLVs. For this experiment, a relatively high concentration of streptavidin (0.1 µM in PBS) was used to completely block the binding sites. The majority of the streptavidin thus would not participate in the binding event. Figure 14b shows SWV measurements for the change of current density on both electrodes (dash lines: before streptavidin–biotin binding, solid lines: after streptavidin–biotin binding). For the negative control, the electrode with non-biotinylated FLVs was used to measure non-specific binding of streptavidin to the FLVs or the gold electrode.

### Electrochemical nanobiosensor: Immunosensor

**Fig. 15:**
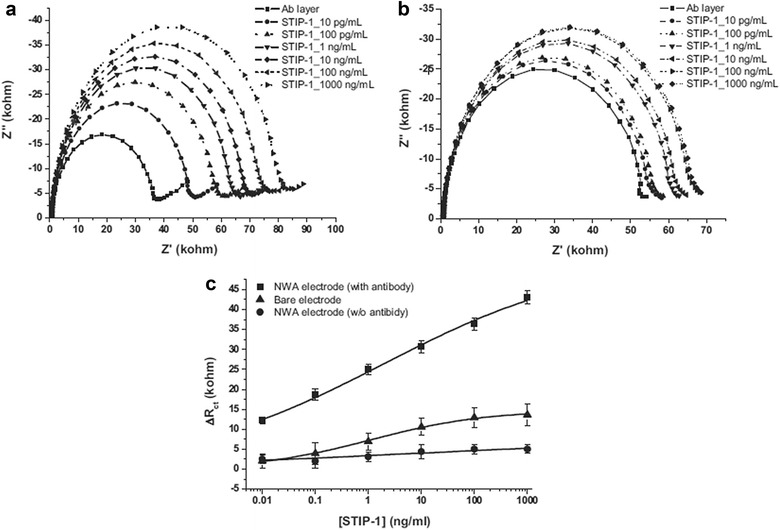
EIS binding of STIP-1 to the immunolayer on the electrodes. **a** Nyquist plot for the NWA electrode. **b** Nyquist plot for the bare electrode. **c** Quantitative analysis of STIP-1 and anti-STIP-1 antibody binding on the NWA and bare electrodes [76]

Figure 15 shows the Nyquist plot for NWA electrodes using STIP-1 as a model analyte. In a nyquist representation, the real component of the complex impedance is shown on the x-coordinate, and the imaginary component on the y-coordinate. The semicircular plot in Fig. 15a, b is typically seen in simple electrochemical systems. The diameter of the semicircle corresponds to the charge transfer resistance, which is inversely related to the charge transfer rate at the interface [77]. By using impedance analysis program (Z plot), we applied the modified randles circuit (R_sol_)(C_dl_[C_ps_R_ct_]) to experimental data, and calculated the R_ct_ component. R_sol_ is the solution resistance, C_dl_ is the double layer capacitance, C_ps_ is the pseudo capacitance and R_ct_ is the charge transfer resistance.

We determined the charge transfer resistance (R_ct_) of the anti-STIP-1 antibody layer to be 36.96 kΩ. After antigen treatment, the R_ct_ was determined to be 48.39, 57.26, 62.98, 68.26, 75.13 and 80.70 kΩ, for 10 pg/mL, 100 pg/mL, 1 ng/mL, 10 ng/mL, 100 ng/mL, and 1000 ng/mL STIP-1, respectively, (Fig. 15a). To compare the sensitivity of the NWA biosensor with that of other electrode-based biosensors, the same treatments were applied to a bare electrode without NWA. For the bare electrode (Fig. 15b), the base R_ct_ was 52.52 k Ω, which represents an increase of 42% over the base R_ct_ of the NWA electrode. The R_ct_ values for 10 pg/mL, 100 pg/mL, 1 ng/mL, 10 ng/mL, 100 ng/mL, and 1000 ng/mL STIP-1 were 54.22, 55.34, 59.94, 61.64, 65.43 and, 65.83 kΩ, respectively.

The differences in the impedance spectra for each electrode before and after STIP-1 addition are shown in Fig. 15c. For the bare electrodes, the impedance at low frequencies interfered with the measurement of the limit of detection (LOD). This low signal-to-noise ratio (S/N ratio) was caused by non-specific binding of STIP-1. It is reasonable to estimate an LOD of 1 ng/mL for the bare electrodes. The LOD was estimated to be 10 pg/mL or less for the NWA electrodes, which suggests a 100-fold improvement in the LOD when using EIS with NWA electrode. The sensitivity of the NWA impedemetric immunosensor was better for each analyte concentration tested when compared the sensitivity of the bare electrode sensor. Therefore, bare electrode has a larger area (4 mm × 2 mm) than NWA electrode (1.75 mm^2^). Basically, electrodes with a larger area provide the sensor with a better S/N ratio because number of binding site is increased. However, in this study NWA’s S/N ratio was higher than the bare electrode although NWA had a smaller area than the bare electrode. The signal for the negative control (without antibody) was not significant, which further supports our finding of high specificity for the NWA electrodes.

Based on these results, the electrochemical impedemetric immune-sensors using NWA electrodes can be applied for label-free detection, with low levels of non-specific binding. Because NWA electrodes are optimal for the selective docking of single molecules, they reduce non-specific binding and enhance electrochemical responses. Therefore, NWA has high sensitivity and selectivity as well as very low LOD.

As previously mentioned, we applied modified randles circuit to fit our experimental data. In the Bode plot, there was a change of the magnitude of the impedance (|Z|) at the low frequency (< 100 Hz). Moreover, the signal in this region was dominated by the dielectric behavior of the electrode. For the high frequency region (> 104 Hz), the resistive region appears and resistance in the solution determines the signal [78–81]. In low frequency region, the magnitude of impedance increases with increasing STIP-1 concentrations, but the impedance change is negligible in high frequency region. The R_ct_ increased by 66.77% for 1000 ng/mL STIP-1, over the value obtained with 10 pg/mL STIP-1. In contrast, the R_s_ increased by only 8.78%, which indicates that R_s_ does not reflect the STIP-1 concentration. This means that higher concentrations of STIP-1 cause increased binding of STIP-1 to the anti-STIP-1 antibody layer, which creates a denser and thicker electrical isolation layer [82–87]. These properties were observed in the low frequency region by EIS. Changes of the double layer properties at lower frequency would be more dramatic than the changes in the solution resistance at the higher frequency region.

## Conclusion

In summary, we have demonstrated that a simple nanowell array (NWA) assay, where the small size of the NWs within the array permits a small quantity of probing biomolecules to be attached to an exposed, can significantly enhance the sensitivity and reliability of detection method for electrochemical biomolecules. The NWA structure has several important advantages. First, it can be used not only for electrochemical detection, but also for the detection of other biomolecules. Second, its application can be extended to other types of biosensors, including DNA, proteins, small molecules and enzyme sensors. Third, it can be easily adapted for simultaneous detection of multiple targets. Finally, it is highly compatible with advanced semiconductor technologies in current use. Therefore, NWA assays can be easily integrated into analytic chips on planar semiconductor substrates. Moreover, we also have presented a NWA based biosensing platform for electrochemically detecting biological reactions on the level of single lipid vesicle that was laden with multiple surface moieties such as biotin, thiol, and ferricyanide. It turned out that the NWA geometry of a gently sloped vertical wall was optimal for selective docking of single liposomes without capillary resistances. The use of a hydrophilic, non-biofouling PEG copolymer was essential to render distinctively separated FLV arrays without non-specific adsorption. With this NWA electrode, the electrochemical responses were significantly enhanced for the binding event of streptavidin to the biotinylated FLVs (~ 220 times increase in signal amplification as compared to bare electrode) and the electron transfer was efficiently blocked by the captured liposome. Also, a simple sandwich format was used for the detection of a specific target of leptin molecules using the NWA electrode. A substantial decrease in the peak current density was found with the addition of leptin without non-specific binding or false positives. It is envisioned that these features could make the molded NWA electrode presented here especially useful for various biological assays involving electrochemical detections. Table 1 is the NWA effectiveness compared with conventional method (ELISA) by using model biomarker (ALP: alkaline phosphatase).

**Table 1:** NWA effectiveness compared with conventional ELISA method

Method	ELISA_fluorescence expression	Electrochemical reaction
Basic principle	Indirect	Direct redox reaction
Limit of detection	0.2 × 10−^2^ U/L (μmol/L)	0.13 × 10−^4^ U/L (μmol/L)
Sample volume	200 μL per test	5 μL per test
Analysis time	1 h	3 min
Detection range	Narrow (10 ng–1 μg/mL)	Wide(10 pg–10 μg/mL)

Alkaline phosphatase (ALP) was used as model biomarker

In addition, a highly sensitive electrochemical impedimetric immunosensor based on wafer-scaled NWA was successfully applied for quantitative detection of various proteins. At low frequencies, binding of protein to the immuno-affinity layer on the NWA electrodes resulted in large changes in impedance, and this effect was not observed at high frequencies. Thus, double-layer properties are more useful than solution resistance for qualifying protein. From the NWA electrodes, we calculated the change in R_ct_ and estimated LOD for specific protein as stress induced phosphoprotein-1 (STIP-1) to be 10 pg/mL which represents a ≥ 100-fold improvement over the bare electrodes which are an electrode without NWA. Electrochemical impedimetric NWA biosensor can also be applied for label-free detection without non-specific binding by means of the selective docking of immobilized antibodies. These properties provide high sensitivity and selectivity for wafer scaled NWA sensor. The results also show the advantages of using NWA over large areas to improve performance and decrease costs. We anticipate that the in vitro nanobiomedical device will lead the way for the realization of digitized nanomedicine at the molecular level.
